# Repair of Damaged Articular Cartilage: Current Approaches and Future Directions

**DOI:** 10.3390/ijms19082366

**Published:** 2018-08-11

**Authors:** Ekaterina V. Medvedeva, Ekaterina A. Grebenik, Svetlana N. Gornostaeva, Vladimir I. Telpuhov, Aleksey V. Lychagin, Peter S. Timashev, Andrei S. Chagin

**Affiliations:** 1 Institute for Regenerative Medicine, Sechenov University, 8-2 Trubetskaya St., Moscow 119991, Russia; grebeneka@gmail.com (E.A.G.); svetlana.gornost@gmail.com (S.N.G.); telpuhov@mail.ru (V.I.T.); timashev.peter@gmail.com (P.S.T.); 2 Department of Trauma, Orthopedics and Disaster Surgery, Sechenov University, 8-2 Trubetskaya St., Moscow 119991, Russia; dr.lychagin@mail.ru; 3 Department of Polymers and Composites, N. N. Semenov Institute of Chemical Physics, 4 Kosygin St., Moscow 119991, Russia; 4 Institute of Photonic Technologies, Research Center “Crystallography and Photonics”, RAS, 2 Pionerskaya St., Troitsk, Moscow 142190, Russia; 5 Department of Physiology and Pharmacology, Karolinska Institutet, Biomedicum 6D, Stockholm 17177, Sweden

**Keywords:** articular hyaline cartilage, regenerative medicine approaches, stem cells, tissue-engineered constructs, cell-based therapy, micro-fracture, mosaicplasty, autologous chondrocyte implantation (ACI), matrix-induced autologous chondrocyte implantation (MACI)

## Abstract

Articular hyaline cartilage is extensively hydrated, but it is neither innervated nor vascularized, and its low cell density allows only extremely limited self-renewal. Most clinical and research efforts currently focus on the restoration of cartilage damaged in connection with osteoarthritis or trauma. Here, we discuss current clinical approaches for repairing cartilage, as well as research approaches which are currently developing, and those under translation into clinical practice. We also describe potential future directions in this area, including tissue engineering based on scaffolding and/or stem cells as well as a combination of gene and cell therapy. Particular focus is placed on cell-based approaches and the potential of recently characterized chondro-progenitors; progress with induced pluripotent stem cells is also discussed. In this context, we also consider the ability of different types of stem cell to restore hyaline cartilage and the importance of mimicking the environment in vivo during cell expansion and differentiation into mature chondrocytes.

## 1. Introduction

Hyaline articular cartilage tissue is extensively hydrated, but it is neither innervated nor vascularized, and its very low cell density allows, unlike bone, only extremely limited self-renewal. Thus, in vivo restoration and/or in vitro reconstruction of hyaline cartilage is the goal of numerous tissue-engineering approaches; however, success remains limited to date.

**Figure 1:**
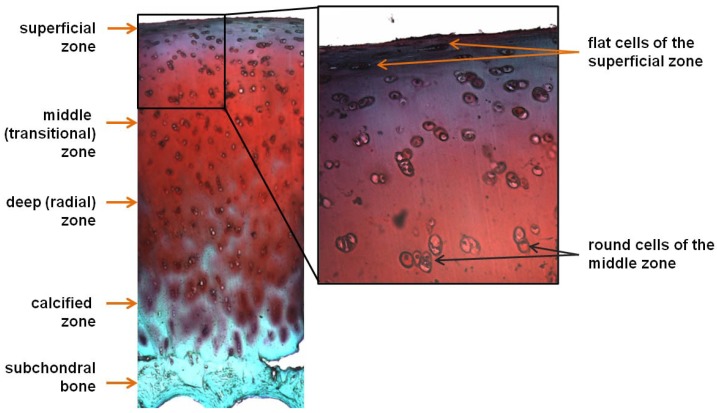
Structure of human articular cartilage. Articular cartilage from a 57-year-old man is stained with Safranin O/Fast Green (images were made by E. V. Medvedeva).

The apparent structural simplicity of hyaline cartilage is deceptive. Despite lacking innervation and blood vessels, this tissue consists of several layers, differing slightly in organization (e.g., cell density, composition of the extracellular matrix (ECM), and orientation of collagen fibers [1]), and thereby, in local elastic modulus [2]. Moreover, although the cartilage contains only a single type of cell referred to as chondrocytes, the cells in different layers have distinct morphologies and functionalities [3]. This tissue is usually divided into four zones: (i) the superficial zone in contact with the synovial fluid, containing chondro-progenitors [4,5]; (ii) the middle or transitional zone beneath the superficial zone, containing round chondrocytes; (iii) the deep or radial zone; and (iv) the calcified layer in direct contact with the underlying subchondral bone (Figure 1).

**Figure 2:**
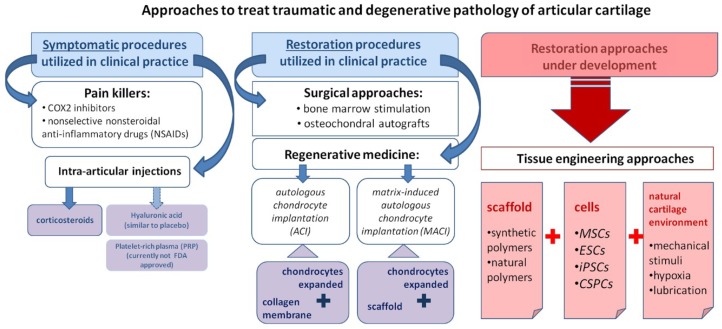
Illustration of approaches to the restoration of cartilage. Abbreviations: autologous chondrocyte implantation (ACI); matrix-induced autologous chondrocyte implantation (MACI); mesenchymal stem cells (MSCs); embryonic stem cells (ESCs); induced pluripotent stem cells (iPSCs); chondrogenic stem/progenitor cells (CSPCs).

Degenerative lesions of articular cartilage as a consequence of destructive joint disease, such as osteoarthritis (OA), can lead to disability, pain during movement of the joint, and gradual deformation of the bone articulation. OA is the most common musculoskeletal disorder, affecting 10–12% of the global population [6]. For people above 65 years of age, this incidence rises to 49.7% (World Health Organization (WHO) statistics 2010), and these numbers continue rising in connection with the aging of the society and an escalating epidemic of obesity. Current treatments of knee and hip OA include cyclooxygenase 2 (COX-2)-selective [7] and nonselective nonsteroidal anti-inflammatory drugs (NSAIDs), as well as intra-articular injections of corticosteroids [8,9], thereby focusing on reducing pain and inflammation without addressing the underlying causes, which eventually leads to joint replacement surgery. The etiology of OA is not yet understood completely; however, aging, trauma, genetic predisposition, obesity, inflammation, and the metabolic syndrome are known to be involved in this disease [10]. The unclear etiology of OA and increased level of inflammation pose additional barriers for regenerative approaches aiming to cure the disease, and most clinical and research efforts in this area currently focus on the restoration of traumatic damage to cartilage, which, if untreated, leads ultimately to the development of OA and the necessity for joint replacement. In this review, we summarize and discuss present approaches to cartilage repair, as well as potential new directions (Figure 2).

Here, we categorized the therapeutic approaches for treating traumatic and degenerative pathology of articular cartilage into three major groups: symptomatic treatment (left-hand side), clinically available restoration procedures (middle column), and those under development (right-hand side). Symptomatic procedures can be further sub-divided into systemic treatment (usually pain killers and anti-inflammatory drugs) and local intra-articular injections, such as injections of corticosteroids or platelet-rich plasma. Clinically available cartilage repair (middle column) can be divided into two sub-categories: surgical approaches (e.g., microfracture and mosaicplasty) and those based on regenerative medicine (e.g., implantation of expanded autologous chondrocytes). The wide variety of approaches to restoration under development (right-hand side) involve cell expansion and differentiation into mature chondrocytes with different combinations of scaffolding, stem cells, and native cartilage environment. 

## 2. Clinically Used Approaches

### 2.1. Intra-Articular Injections of Various Compounds

Intra-articular injection is a minimally invasive procedure used to directly deliver compounds to a specific joint. As intra-articular injections can be performed easily in an outpatient setting, this approach is used to test the efficacy of many compounds for OA treatment. Below, we briefly summarize the most common compounds administered via intra-articular injection (see also Figure 2). 

#### 2.1.1. Corticosteroid Injections

The Osteoarthritis Research Society International (OARSI) guidelines recommend intra-articular injections of corticosteroid as an anti-inflammatory agent to reduce joint pain (arthralgia) [11]. Similarly, the United Kingdom (UK) National Institute of Care Excellence (NICE) and American College of Rheumatology (ACR) consider intra-articular corticosteroid injections as an adjunct to core treatments for the relief of joint pain in patients with OA [12,13]. The beneficial effect occurs at low doses, whereas high doses and prolonged exposure are associated with significant gross cartilage damage and chondrocyte toxicity [14], and are even shown to accelerate the progression of OA [15]. An analysis of multiple time-points suggests that the efficacy of corticosteroid injections is reduced over time [16].

#### 2.1.2. Hyaluronic Acid (Hyaluronan) Injections

Hyaluronic acid (or hyaluronan, HA), a non-sulfated glycosaminoglycan, is a critical component of normal synovial fluid and an important contributor to joint homeostasis [17]. In OA, the concentration of HA in synovial fluid is often diminished and its molecular weight is decreased due to dilution, fragmentation, and the synthesis of shortened HA polymers [18]. Intra-articular HA injections are used for so-called viscosupplementation therapy, which is based on the concept of replenishing the HA toward normal levels of molecular weight and concentration [19,20]. Intra-articular HA injections received United States Food and Drug Administration (FDA) approval 20 years ago. However, a meta-analysis of randomized clinical trials did not find a significant effect of intra-articular injections of HA in the treatment of OA compared with intra-articular injections of a placebo [21,22,23].

#### 2.1.3. Injections of Autologous Platelet-Rich Plasma

Platelet-rich plasma (PRP) is an autologous blood product containing highly concentrated platelets and various types of growth factors, proteases, and cytokines, which are thought to activate a variety of signaling pathways promoting tissue repair [24,25,26]. A proteomic profile analysis of isolated human platelets identified more than 1500 unique proteins [26,27].

The majority of studies looking at the use of PRP intra-articular injections in degenerative OA report improvements in pain and functional outcome scores [28] with no studies reporting worsening scores [25]. Plasma concentrations of inflammatory and pro-angiogenic factors were significantly alleviated in patients receiving PRP as compared with the placebo group [29]. However, the mechanism of PRP action in arthritic joints is unknown [24].

Currently, PRP injections are not approved by the FDA and are not recommended by the OARSI for OA treatment due to the lack of conclusive and reliable clinical evidence. Additionally, high-quality long-term data are also lacking [25].

### 2.2. Surgical Approaches: Microfracture and Chondroplasty Surgery

Microfracture [30] and similar techniques (i.e., abrasion [31] and drilling [32,33,34]) involve disrupting the subchondral bone integrity to create channels between the defect in the cartilage and underlying bone marrow (Figure 2). It is generally accepted that the recruitment of multi-potent marrow stromal cells to the defect through these channels leads to subsequent formation of tissue resembling articular cartilage. However, this approach is only effective for small defects [35], and moreover, provides relatively short-term functional improvement due to the formation of fibrocartilage rather than hyaline articular cartilage [36]. Nevertheless, these techniques are used widely because of their simplicity and low cost.

Another surgical procedure involves the replacement of the lost cartilage with tissue grafts, i.e., an osteochondral allograft [37] or autologous transplant harvested from the patient’s own cartilage (referred to as mosaicplasty [38]; Figure 2). In the latter case, small cylindrical plugs taken from non-weight-bearing areas are fitted into the defect (Figure 2) [32,33]. Although restoration of the defect via mosaicplasty often produces a desirable functional outcome, the results can vary greatly depending on age, sex, and size of the lesion [39]. Other drawbacks include donor-site soreness and limited availability of donor tissue, rendering mosaicplasty applicable only to small and certain intermediate-size defects [40]. In addition, mosaicplasty is surgically challenging, since all the plugs implanted must be adjusted to provide an even cartilage surface. The challenges associated with osteochondral allograft transplantation include proper storage of the allograft, tissue availability, the possibility of an immunologic response by the recipient, and demanding surgery [41].

### 2.3. Regenerative Medicine and Cell-Based Approaches

**Figure 3:**
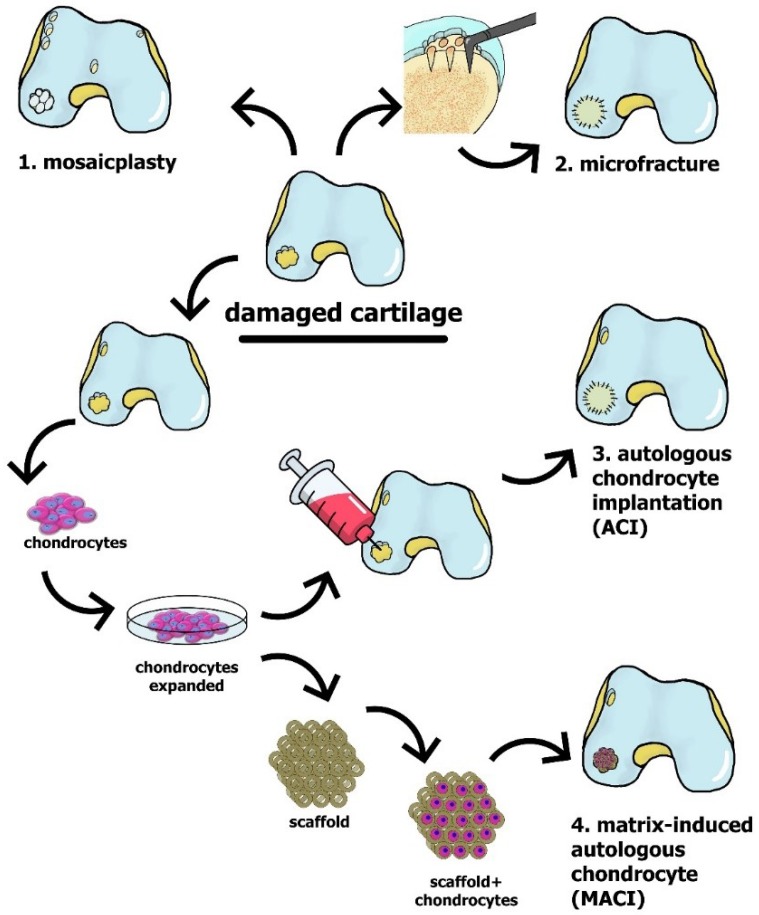
Illustration of the clinically approved approaches to restoration of cartilage tissue.

The first approach to cartilage regeneration, autologous chondrocyte implantation (ACI) (Figure 2 and Figure 3), was developed by Brittberg and colleagues in 1994 [42] and involves harvesting small pieces of the patient’s own cartilage, followed by the expansion of chondrocytes in the laboratory and subsequent injection of the cultured chondrocytes into the defect. The cells injected were originally covered with an autologous periosteal patch harvested from the bone (initial ACI [42]), which prevents the outflow of injected cells into the joint cavity and facilitates the formation of new tissue [43]. Subsequently, in second-generation ACI, biodegradable collagen membranes replace the periosteal patch [43,44], avoiding the invasiveness of periosteal harvesting and the extensive chondrocyte hypertrophy that sometimes occurs in association with the periosteum [45]. Compared to microfracture or mosaic chondroplasty, ACI allows repairs of larger cartilage defects [46,47]. The main limitations to this approach include its high cost [48,49], as well as the invasiveness of harvesting, and, in particular, the formation of fibrocartilage, which often occurs due to the de-differentiation of chondrocytes during cell expansion [44]. Interestingly, in the case of small-to-intermediate-sized cartilage defects, ACI and microfracture provide comparable clinical outcomes [50], whereas when the subchondral bone is disrupted by a prior surgery or fracture, osteochondral allografts are often the better choice [4,33].

In hyaline cartilage, the chondrocytes reside within an extracellular matrix rich in collagen fibers that support tensile strength, as well as within proteoglycan complexes that provide compressive strength [51]. Thus, the development and clinical implementation of matrix-based cell therapy of cartilage defects, matrix-induced autologous chondrocyte implantation (MACI) (Figure 2 and Figure 3), was the logical extension of ACI [32,44]. This procedure involves transplantation of a special three-dimensional scaffold comprised of autologous chondrocytes (expanded previously) into the cartilage defect. During the first two years after surgery, satisfactory results were obtained with both MACI and microfracture, but an improvement with MACI was significantly better five years post-surgery [47].

The range of techniques widely used for cartilage restoration in clinic practice include the following: (1) mosaicplasty—the replacement of lost cartilage with an autologous transplant harvested from a non-weight-bearing area of the articular cartilage; (2) microfracture—the disruption of subchondral bone to promote recruitment of multi-potent bone-marrow-derived stromal cells to the cartilage defect; (3) ACI—the in vitro expansion of autologous chondrocytes harvested from a non-weight-bearing area of the articular cartilage and subsequent injection of these cells into the defect, covering them with a biodegradable collagen membrane; and (4) MACI—the transplantation of a commercial scaffold containing autologous chondrocytes expanded previously.

A large number of commercial products for the implementation of this method and its modifications are already available. These are mainly expanded autologous chondrocytes seeded onto different types of scaffolds that mimic the mechanical properties of the matrix of native articular cartilage, such as the bilayer collagen type I/III scaffold (MACI), honeycomb bovine type I collagen scaffold (NeoCart^®^), bilayer type I collagen sponge containing chondroitin sulfate (NOVOCART^®^ 3D), mesh of hyaluronic-acid-based microfibers (Hyalograft^®^ C), and agarose/alginate hydrogel (Cartipatch^®^). Scaffold-free (endogenous scaffold-based) spheroids of autologous cells (Chondrosphere^®^) and neocartilage discs composed of allogeneic juvenile chondrocytes (RevaFlex™) are also available (reviewed in Reference [52]).

Although the implantation of mature cultured chondrocytes is performed worldwide, there are still unresolved challenges associated with the maintenance of these chondrocytes in a stable state. The expansion of autologous chondrocytes in vitro to obtain a sufficient number of cells is invariably associated with chondrocyte de-differentiation [53], reduction in the expression of cartilage-specific type II, IX, and XI collagens, as well as aggrecans (ACANs) [54] and glycosaminoglycans (GAGs), and elevated synthesis of non-specific type I collagen [55]. Accordingly, such cells often develop into fibrocartilage rather than the hyaline cartilage desired. On the other hand, mature differentiated chondrocytes do not proliferate, and cannot, therefore, be easily expanded in vitro [55]. Thus, maintenance of the appropriate chondrogenic phenotype and the ability to proliferate are mutually exclusive.

Numerous research efforts focused on finding a balance between these two states, employing various differentiation strategies. Dulbecco's modified Eagle’s medium (DMEM) and DMEM/F12 culture media are commonly utilized for the expansion of chondrocytes either with or without serum. Additionally, 10–20% fetal bovine serum, allogenic serum, or autologous serum is commonly used for the ACI/MACI procedure, whereas three-dimensional (3D) cultures are usually serum-free. Serum-free conditions eliminate the risk of disease transmission from animal products, immunogenic issues, potential adverse effects on the cell’s chondrogenic potential, and the inconsistency associated with the use of serum, which cannot be standardized. However, serum-free medium must be supplemented with growth factors, most commonly fibroblast growth factor 2 (FGF-2 or bFGF) and transforming growth factor-β1 (TGF-β1) individually or in combination [56,57]. In 3D cultures (pellet culture, alginate encapsulation, suspension culture, culture within a scaffold, etc.) chondrocytes can grow for months with a preserved phenotype [58,59].

Another approach to overcoming de-differentiation is to minimize the number of passages, which varies. For example, in the case of MACI, chondrocytes are used up to passage 3 (P3), whereas, for other bio-engineered products, this can range from P0 to P4 passages. Although gene expression changes drastically upon prolonged cultures, no difference in clinical outcome was reported [52,60]. Chondrocyte re-differentiation can be promoted using various strategies, such as the supplementation of bone morphogenetic protein 2 (BMP-2), 3D cultures, small interfering RNA (siRNA) transfections [61], and high-density [62] as well as low-density culture [63]. However, after many (>4) passages chondrocytes lose their ability to re-differentiate partially or completely [62]. In addition to this problem with de-differentiation, the proliferative capability of chondrocytes appears to decrease with the age of the donor [64] which can obviously limit their use for ACI/MACI.

Thus, the proper balance between chondrocyte proliferation and differentiation is yet to be fully achieved. Another strategy would involve using an alternative cell type that does maintain its inherent proliferative capacity, such as mesenchymal stem cells (MSCs), induced pluripotent stem cells (iPSCs), chondrocyte stem/progenitor cells (CSPCs), etc. This approach has the additional advantage of avoiding invasion of the joint for initial harvesting of chondrocytes.

## 3. Regeneration of Cartilage with Stem Cells

### 3.1. Mesenchymal Stem Cells

Mesenchymal stem cells (MSCs) from different sources, such as the bone marrow, adipose tissue, synovial membrane, cord blood, periosteum, and muscle, are employed to treat defects in articular cartilage [65,66]. Indeed, the easy availability, extensive potential for differentiation and proliferation, and anti-inflammatory and immunomodulating properties [67] of these cells are promising in connection with cell therapy. The ability to differentiate into chondrocytes varies between MSCs obtained from different sources, with synovial MSCs demonstrating the greatest potential to differentiate into articular chondrocytes [68]. However, the transplantation of MSCs often gives rise to a mixture of hypertrophic, cartilaginous, and fibrous tissues, which is not particularly sustainable, and, in the long run, leads to a loss of repair tissue [69]. Thus, a further development of culture/differentiation protocols is required before MSCs can be utilized successfully for joint repair.

### 3.2. Embryonic Stem Cells

Embryonic stem cells (ESCs) possess unlimited potential for proliferation and differentiation into virtually any type of somatic cell [70,71,72]. The various procedures for the conversion of ESCs into chondrocytes include co-culture with primary articular chondrocytes [73,74] and the production of cells resembling mesenchymal stem cells from ESCs, followed by their differentiation into chondrocytes employing a variety of growth factors [72,75]. The most successful differentiation of ESCs into chondrocytes involves differentiation-mimicking embryonic development, i.e., the induction of primitive streak cells with BMP4 and bFGF, followed by the generation of paraxial mesoderm via the inhibition of BMP signaling in the presence of bFGF, the generation of chondrocyte progenitors in high-density culture in the presence of TGF-β3, and the production of articular chondrocytes with time [76,77].

The drawbacks associated with the utilization of ESCs for cartilage regeneration include ethical concerns about the destruction of a human embryo, immune rejection by the host, poor survival of human ESCs following disintegration of the cell mass, and the risk for teratoma formation [78].

### 3.3. Induced Pluripotent Stem Cells

Induced pluripotent stem cells (iPSCs) represent a relatively new source of stem cells with the capacity for self-renewal and pluripotency similar to that of ESCs, but without the same ethical and immunogenic concerns. The iPSCs are obtained by reprogramming somatic cells in vitro to enter an embryonic-like pluripotent state through the introduction and forced expression of the four transcription factors (TFs)—octamer-binding TF 4 (Oct4), sex-determining region Y (SRY)-box 2 (Sox2), cMyc, and Krüppel-like factor 4 (Klf4) [79], referred to collectively as Yamanaka factors. Although these cells can be generated from many different types of somatic cells, skin fibroblasts are the major source because of the ease with which they can be obtained. However, the efficiency in this case is relatively low, with less than 1% of transfected fibroblasts becoming iPSCs [80]. Furthermore, iPSCs can also be derived from keratinocytes, mesenchymal cells, adipose stem cells, melanocytes, and postmitotic neurons [80].

The strategies and procedures for generating chondrocytes from human iPSCs (hiPSCs) are currently being developed and improved extensively [77,81,82,83]. Among the various approaches for inducing the chondrogenic differentiation of human ESCs currently applied to iPSCs, the most promising mimic natural development, with monolayer cultures of iPSCs (or ESCs) first differentiating into the mesoendoderm, followed by further differentiation into chondrogenic cultures [84]. The steps in this process vary slightly between laboratories; however, in general, they include the modulation of BMP/TGF-β, FGF, and Wingless-type MMTV integration site (Wnt) signaling pathways, as well as alterations in culture conditions, such as the monolayer cell density, two-dimensional (2D) versus 3D culture, etc. [76,84,85,86]. However, the purity and homogeneity of the newly formed cartilage still vary, and the in vivo transplantation of chondrocytes derived from hiPSCs still raises concerns about tumor formation [87,88], although the first clinical application of hiPSCs for the treatment of macular degeneration resulted in no signs of carcinogenesis [89].

At the same time, this recent study revealed an unprecedentedly high cost for the clinical application of iPSCs derived from the patient, due to the extensive validation required, e.g., whole-genome sequencing of several cell lines obtained, as well as their testing in vitro. An alternative strategy, proposed by Prof. Yamanaka and currently being developed in several countries, involves the generation of a number of iPSC cell lines from so-called “superdonors” (donors homozygous for the most common human leukocyte antigen (HLA) alleles) to sufficiently encompass immunological variety [90,91]. In the same way that recipients of organ transplant are paired with immunologically compatible donors through HLA matching, Yamanaka is now establishing a bank of HLA-homozygous iPSCs that covers most of the Japanese population [91]. It is estimated that just 100 cell lines homozygous for the most common HLA types in each population would match approximately 78% of Northern Europeans, 63% of Asians, 52% of Hispanics, and 45% of African Americans [90]. One hundred and forty HLA-homozygous iPSC cell lines are estimated to cover 90% of the population of Japan (Prof. Yamanaka’s public lectures). This approach should improve engraftment, with a lower immune response and greater survival of the transplanted cells [92]. Thus, in theory, a bank of validated and ready-to-use iPSC cell lines with well-characterized HLA could be used to generate chondrocytes for the repair of articular cartilage.

### 3.4. Chondrogenic Stem/Progenitor Cells from the Superficial Zone

In 2004, the existence of chondrogenic stem/progenitor cells (CSPCs) in the superficial zone of bovine articular cartilage was proposed on the basis of their adhesion to fibronectin, expression of stem-cell markers, extensive proliferative capacity, and ability to differentiate into chondrocytes in vitro [93]. Recently, several research groups employed genetic tracing to confirm the presence of CSPCs in the superficial zone of murine articular cartilage [94]. These CSPCs can be expanded extensively in vitro [93], form the entire adult articular cartilage in vivo [4], and likely contribute to the physiological healing of small defects in cartilage [95].

High therapeutic potential of CSPCs in connection with articular cartilage repair was indirectly supported by the recently observed superiority of autologous CSPC-derived cartilage over that obtained with autologous chondrocytes [96]. However, certain issues remain to be resolved. The definitive identification and purification of CSPCs from adult human articular cartilage is difficult due to the lack of well-defined markers, and current approaches are based on their high adhesion to fibronectin [96]. In addition, the therapeutic potential of these cells is yet to be tested in either animals or humans.

Thus, each source of cells has its own advantages and drawbacks, and an additional evaluation of their potential, and, in particular, their long-term outcomes is required.

## 4. Tissue-Engineered Constructs

### 4.1. Scaffolds

Tissue engineering for the restoration of damaged articular cartilage involves several different scenarios. The basic scenario utilizes synthetic or natural scaffolds that mimic the ECM of native cartilage. In an advanced scenario, tissue-engineered constructs are loaded with living cells and/or growth factors which facilitate the integration of the implant into the host tissue. Scaffold-free products are presented only by the condensed spheroids of chondrocytes obtained from articular cartilage, which are available commercially under the trademark Chondrosphere^®^ (co.don^®^ AG, Berlin, Germany) [97]. The following section focuses on scaffold-based approaches.

The polymers utilized for the tissue engineering of articular cartilage are both synthetic and natural. Natural polymers are limited to alginate [98,99,100,101], gelatin [102,103], agarose [104,105], hyaluronic acid [106], fibrin, and collagen [107]. The synthetic group is more diverse and generally includes poly(*ɛ*-caprolactone) [102,108], poly(l-lactic acid) [109,110], poly(lactic-co-glycolic acid) [111,112], poly(vinyl alcohol) [113], polyethylene glycol [114], pluronics [115], polyurethane [116], and self-assembling peptides [104]. Natural polymers are both biodegradable and biocompatible, but their composition varies from batch to batch. Synthetic polymers are more easily reproducible, with properties that can be precisely controlled [103,104,105,109,110,111,112,113,114,115,116,117]. Among others, scaffolds based on polycaprolactone [118] and self-assembling peptides [119] were shown to sustain the proliferation and differentiation of chondrocytes in vitro. Nonetheless, natural polymers are most widely used in ongoing clinical studies, with collagen being the most common. Collagen scaffolds provide the foundation for autologous matrix-induced chondrogenesis, both cell-free [120,121,122] and cell-assisted [120,123]. Of particular interest is MACI aided by collagen [124,125,126], hyaluronic acid [127,128], or fibrin glue [129].

### 4.2. Production of Scaffolds

The polymers employed for the scaffolds must exhibit tissue-like mechanical properties, biocompatibility, and resistance to wear. These scaffolds are produced using various techniques, including freeze-drying [130], molding [131], electrospinning [107,132], 3D bio-printing [99], and stereolithography [133], sometimes with the aid of a specific material (e.g., poly(vinyl alcohol) or alginate) that serves as a temporary mold or porogen. Subsequent leaching of this temporary material provides the scaffold with a complex architecture and enhanced porosity [108,134,135,136] that support the chondrogenic differentiation of MSCs [109]. Although porogen leaching is one of the most accessible, this process is complicated by the limited number of appropriate porogen-solvent combinations, mechanical properties that are inadequate for load-bearing applications (due to the highly porous structure), uneven pore density, and the presence of residues of organic solvent in the scaffold.

Electrospun nanofibrous scaffolds are composed of ultra-fine biodegradable polymers, most commonly poly(α-hydroxyesters) [110,137]. The applicability of nanofiber scaffolds seeded with MSCs was demonstrated for the tissue engineering of articular cartilage both in vitro and in vivo [113,138].

The extent of scaffold-assisted chondrogenesis is commonly assessed on the basis of an increase in the content of sulfated glycosaminoglycan (GAG) and the expression of collagen type II and aggrecan [139]. Natural polymers, such as collagen [111], silk fibroin [140], fibrin [141], chondroitin sulfate, or hyaluronic acid [142], are often included in synthetic scaffolds to enhance chondrogenic differentiation. Of special interest in this context are self-assembling peptides, which are compatible with chondrocytes and do not require chemical or thermal treatment in order to form a scaffold [104,112,143]. For example, chondrocytes cultured within a hydrogel of RAD-16 self-assembly peptide (Ac-RADARADARADARADA-CONH2) produced GAG and type II collagen extensively [143].

### 4.3. Three-Dimensional Bio-Printing

Layer-by-layer 3D bio-printing based on computer-aided design (CAD) allows the construct to be customized to the shape of the individual defect [108]. Bio-printing of cartilage constructs is generally extrusion-based, although the resolution of the fiber thickness is limited to ~100 µm. Alternatively, inkjet [144] and laser-induced forward-transfer (LIFT) [101] 3D bio-printing provide greater resolution, but are quite expensive.

The use of hydrogel-based bio-inks enables the homogenous incorporation of cells and biological factors during production, while retaining mechanical support [103,145]. Importantly, the water content of hydrogels (~80 wt %) is similar to that of articular cartilage. The polymers used in hydrogels are often naturally occurring. Among them, alginate, agarose, and silk fibroin take favor with a low biodegradation rate and compatibility with chondrocytes, although, at the same time, their low adhesiveness and bio-inertness limit the regenerative potential. The bio-ink can also be rendered bioactive by incorporating various functional components [145].

Collagen and hyaluronic acid, inherent components of articular cartilage, support cell attachment and stimulate formation of the ECM, but exhibit little mechanical stability and are subject to intense biodegradation [146,147]. Synthetic polymers are superior to these natural ones in terms of controllable biodegradation and biomechanics, but often demonstrate poor biocompatibility and require modifications to provide specific biological functions. Thus, hybrid bio-inks are often combinations of polymers with different desirable properties [148,149,150].

The gelation of bio-inks is achieved via ionic, thermal, or photo cross-linking, depending on the nature of the polymer present. Ionic cross-linking is applicable to alginate-based constructs, while temperature-induced gelling is best for thermoresponsive polymers (e.g., collagen, agarose), and photo-curing is generally applied to biomaterials modified appropriately with acrylate or methacrylate moieties. These procedures are all well established, but each has its own drawbacks. In particular, ionic cross-linking results in low-resolution bio-printing [144]; photo-initiators are often cytotoxic [151]; and the temperature fluctuations and shear stress during thermal printing may affect cells subsequently incorporated [152]. The mechanical properties of hydrogels can be tailored to mimic those of articular cartilage via the introduction of thermoplastic polymer fibers [98,102] or additional cross-links [115]. Recently, a number of commercially available tissue-engineered constructs, both synthetic and based on natural polymers, demonstrated favorable clinical outcomes [153,154]. However, several limitations still impede the complete and sustained repair of damaged articulate cartilage tissue.

Interestingly, the 3D printing of cartilage constructs shaped like the human ear was recently achieved using a composite hydrogel containing evenly distributed rabbit ear chondrocytes [155]. These elastic cartilage constructs were implanted into the dorsal subcutaneous space of athymic mice, and, for one–two months, the cells in the newly formed tissues within typical chondrocyte lacunae were viable and received adequate nutrients during their maturation [155]. However, 3D bio-printing of more complex zonal cartilage is still a challenging task. Various subpopulations of chondrocytes can be harvested from different zones of cartilage tissue [3], but de-differentiation of expanded chondrocytes and the limited availability and phenotypic instability of isolated chondrocytes still represent insurmountable obstacles [156].

## 5. Approaches Mimicking the Natural Environment of Articular Cartilage

### 5.1. Lubrication

Among other factors, low friction at the joint surface is of considerable importance. Achieving a low coefficient of friction between interfacing cartilage surfaces is facilitated by the expression of lubricin (also known as proteoglycan 4 (PRG4) and as superficial zone protein) [157]. Lubricin, a secretory mucinous glycoprotein encoded by the *PRG4* gene, is produced both by synoviocytes and the superficial cells located in the upper layer of articular cartilage [158], and acts as a lubricant. Lack of PRG4 results in loss of chondrocytes from the superficial and upper intermediate zones of mouse cartilage [159], whereas intra-articular injection of human PRG4 into synovial joints of PRG4-deficient mice prevents caspase-3 activation in the superficial zone [160]. Various lubricin-mimetic molecules (mLub) less vulnerable to enzymatic digestion were developed [1].

Reducing surface friction through the injection of mLub into the joint during the early stages of osteoarthritis suppresses further degeneration of cartilage [161]. Alternatively, friction can be lowered via the stimulation of *PRG4* expression with growth factors [162]. Indeed, cytokines of the TGF-β family stimulate lubricin secretion in both the superficial zone and synoviocytes in a dose-dependent manner [163]. Bone morphogenetic proteins (BMP-2, BMP-4, BMP-7, and growth/differentiation factor 5 (GDF-5)) also upregulate PRG4 expression, more so in synoviocytes than superficial chondro-progenitors [163].

Interestingly, these growth factors promote lubricin synthesis by different types of stem-like cells. Specifically, kartogenin, TGF-β1, and BMP-7 enhance lubricin accumulation in bone-marrow-derived MSCs (BMSCs) [164], in STRO-1- and activated leukocyte cell adhesion molecule (ALCAM (CD166))-positive muscle-derived MSCs (MDMSCs) [165], and in mesenchymal progenitor cells derived from the infrapatellar fat pad and synovium [166,167], but not in human ESCs differentiated toward articular cartilage [168]. Thus, lowering the friction of engineered cartilage, either by injecting mLub and/or promoting the expression of *PRG4*, might improve the outcome of implantation surgery.

### 5.2. Mechanical Stimuli

Proper maintenance of chondrocyte differentiation and the intensity of matrix production depend not only on the scaffold, but also on the environment [169]. It is now generally accepted that mechanical stimuli and hypoxia have a dramatic influence on adult articular cartilage. It was shown that the hindlimb immobilization of rodents results in catabolic changes and cartilage degradation [170]. Mechanical stimulation improves the quantity and quality of cartilage produced [171] and special mechanobioreactors can mimic the cyclic compressive loading and shear forces of the natural joint during cultivation in vitro [172]. Stimulation of cultured chondrocytes by hydrostatic pressure (HP) is beneficial for properties of generated cartilage and employed commercially (0.5 MPa, 0.5 Hz, Neo-Cart^®^ product, Histogenics, Waltham, MA, USA (patent information)). It is important to note that the outcome of such stimulation depends on the regimen, magnitude, frequency, and duration; accordingly, conditions must be optimized for each individual system (e.g., monolayer or 3D engineered constructs). Interestingly, intermittent HP of physiological magnitudes (5–10 MPa) was used to promote the differentiation of MSCs, ESCs, and de-differentiated chondrocytes [173]. Finally, in mice, elevated fluid flow shear stress in combination with running promotes the secretion of PRG4 by superficial cells [174].

### 5.3. Hypoxia

The physiological level of oxygen in adult cartilage is normally low (1–10%). Oxygen tension within cartilage tissue depends on a number of factors, including oxygen concentration in the synovial fluid, distance from the surface of cartilage, thickness, and cell density [175]. In vitro hypoxia promotes the expression of genes encoding constituents of the cartilage matrix, as well as of the key cartilage transcription factor, Sox9, probably by suppressing the degradation of hypoxia-inducible transcription factor (HIF1-α) [176,177]. Low levels of oxygen also slow age-related changes in the composition and structure of the ECM [178]. However, the effect of hypoxia on the expression of *PRG4* by superficial cells is rather controversial [179,180]. Assuming that oxygen is supplied to the joint predominantly via synovial fluid, the superficial zone should be exposed to the highest levels, and indeed, a gradient of oxygen tension exists across the layers of cartilage [178]. Thus, maintenance of a low level of oxygen (mimicking hypoxic conditions of healthy cartilage [176]) may help optimize the culture of cartilage-engineered constructs [178,181].

## 6. Regenerative Approaches for Treatment of Osteoarthritis

As mentioned in the introduction, the etiology of OA is not very clear, and increased levels of inflammation as well as other co-founding factors may impair the efficacy of regeneration strategies described above. As a potential approach, therapeutic strategies with anti-inflammatory properties may serve as a favorable direction [182].

It was shown that MSCs secrete a variety of cytokines and growth factors with immunosuppressive effects [182,183]. Furthermore, MSCs exert an immunosuppressive effect on activated immune cells such as T cells and mast cells [182], and MSC-treated macrophages acquired an anti-inflammatory M2 phenotype [184]. Thus, employing MSCs for cartilage repair during OA may theoretically benefit from their immunomodulatory activity [183,185]. Interestingly, iPSCs have similar immunogenic properties, but more potent immunomodulatory effects than MSCs [186], and chondro-progenitors obtained from human iPSCs exhibited immunophenotypic features of MSCs [187].

Gene-therapy approaches for the anti-inflammatory treatment of OA are also under development [7]. The delivery of target mediators is implemented through the direct intra-articular injection of a plasmid/vector (in vivo gene therapy) or the intra-articular delivery of transduced cells (ex vivo gene therapy) [7,182].

Intra-articular delivery of genes coding soluble interleukin 1 (IL-1) receptor (IL-1Ra), IL-10, TGF-β1, and Sox9 reduced the inflammatory process and promoted the regeneration of cartilage tissue [8,182]. The ex vivo transfection of synovial fibroblasts with an IL-1Ra-expressing vector following their re-implantation prevents leukocyte infiltration and cartilage tissue degradation, and this therapy (sc-rAAV2.5IL-1Ra, Mayo Clinic, Rochester, MN, USA) was approved for a Phase I clinical trial in the United States [7,9]. A similar approach, but with the genetic delivery of TGF-β, known as Invossa^TM^ (TissueGene, Inc., Rockville, MD, USA), was found to promote cartilage repair in a rabbit defect model [188]. Phase II clinical trials demonstrated that Invossa^TM^ is safe and effectively improves pain and motor scores compared to a placebo group in patients with moderate-to-severe disease [189,190]. Recently, Invossa^TM^ was approved in South Korea for the treatment of moderate knee OA, and it is currently in Phase III clinical trials in the United States [7,9]. Recent efforts are also focused on the intra-articular delivery of small regulatory nucleic acids, such as microRNAs (miRNAs) [7]. More than 30 miRNAs expressed in human joint tissue are involved in cartilage homeostasis and OA development [191]. Among those, miRNA-140 was reported as a regulator of anti-inflammatory and pro-anabolic signaling [192], and intra-articular injections of miRNA-140 can alleviate OA progression [193]. Wang et al. (2016) demonstrated that the retrovirus-based delivery of miR-142-3p significantly inhibited the production of pro-inflammatory cytokines [194].

Thus, a combination of gene therapy and regenerative approaches might be a way of combating OA in the future; however, at the current stage, the results are still very preliminary. A better understanding of OA etiology might help developing an optimal strategy in this direction.

## 7. Conclusions

All treatments of defects in joint cartilage have their limitations. The treatment of larger lesions (>4.5 cm^2^) with regenerative approaches (i.e., ACI/MACI) produces more favorable outcomes than with a microfracture [46,47], which is most commonly used at present. However, no current repair therapy re-creates native hyaline cartilage and provides long-term restoration [33,195], due mainly to the formation of fibrocartilage and/or poor matrix properties. Combining different approaches, including advanced scaffolds, efficiently differentiated chondrocytes, 3D printing of engineered constructs, proper lubrication, and approaches affecting the pro-inflammatory milieu, might greatly improve the regeneration of articular cartilage.
